# Fractals for an ethnography of time and addiction: Recursive and self-similar temporalities in heroin and poly-substance use

**DOI:** 10.3389/fpsyt.2023.1116142

**Published:** 2023-02-02

**Authors:** Laura Roe, Sonja Dobroski, Gabriela Manley, Holly Warner, Heidi Dritschel, Alexander Mario Baldacchino

**Affiliations:** ^1^Department of Social Anthropology, School of Philosophical, Anthropological and Film Studies, Faculty of Arts, University of St Andrews, St Andrews, United Kingdom; ^2^Department of Social Anthropology, School of Social Sciences, Faculty of Humanities, The University of Manchester, Manchester, United Kingdom; ^3^Department of Anthropology, Faculty of Social Sciences and Health, Durham University, Durham, United Kingdom; ^4^Edinburgh Futures Institute, The University of Edinburgh, Edinburgh, United Kingdom; ^5^School of Psychology and Neuroscience, University of St Andrews, St Andrews, United Kingdom; ^6^School of Medicine, University of St Andrews, St Andrews, United Kingdom

**Keywords:** addiction, substance use, poly-substance use, time, temporality, fractal, fractal analyses, memory

## Abstract

Drawing on both mathematical and anthropological understandings of fractality, this paper explores alternative perspectives of time as it relates to heroin addiction and poly-substance use in Scotland. The paper ethnographically illustrates temporalities which confound typical conceptualizations of linearity, and which can be better understood as fractal. Senses of linear time are disrupted for people who use heroin through intensive poly-substance use, an increasing trend in Scotland, as both time and memory become fragmented beyond coherence or re-assemblage. Distortedness and complexity being common descriptors applied to mathematical fractals, time shattered into uncountable and un-interpretable fragments similarly connotes fracture, dissonance, and distortion. A meaningful engagement with fractal theory contains the potential to open up new vocabulary, imagery, and theoretical avenues with which to grasp complex and non-linear time experience. The aims of the paper are, therefore, twofold; to both provide a nuanced ethnographic exploration of substance use time, and to develop a reflexive analytical framework for temporal experience through fractals.

## Introduction

Time comprises a burgeoning mode of inquiry in substance use scholarship, as much as its study remains disparate and, in large part, under-explored within the field. Explorations of how time, temporality, and memory are experienced, perceived, reckoned with, and at times actively harnessed, however, lay bare complex experiences of addiction and intimate aspects of everyday life for both people who use drugs and those in abstinence-based recovery (1–3). Temporality, for example, materially impacts how services are engaged with, the diverse ways in which services structure and organize time often being at odds with service users’ own situation in time (2). Reith (4) notably argues that addiction “reorders” time, her participants retrospectively characterizing long periods of drug use as fractious and non-linear, and yet defined by a sense of the present as “extended” or “frozen.” Abstinence-based recovery, in sharp contrast, “reanimated” time, opened up the future, and was typified in linear, spatialized terms of “moving forward” and “looking backwards” (4).

During Laura Roe’s ethnographic fieldwork–on which this paper is based–services, people who used drugs, and those in recovery, consistently employed spatial metaphors while portraying temporalities of drug use, intoxication, and recovery (5). In abstinence, time was framed as a “forward” progression from past, present, to future, although often cyclically looped back on itself or recessed if drug use was returned to. Active polydrug use and intoxication alternatively gave rise to temporal incoherence, fragmentation, and disorder–at times near impossible to express in language. Time was keenly distorted in the high itself, often felt to pause or stretch indefinitely, depending on combinations of drugs taken and physical circumstances. Throughout the fieldwork, questions around how to grapple with such spatio-temporal complexity–and how to account for multitudes of diffuse, shifting, and contradictory experiences that often seemed to sit outside of language–were ever present; questions that this paper aims to address. It does so in part through an engagement with fractal geometry.

Applications of fractal geometry have long occurred outside of mathematics, comprising a prolific area of research across the natural and social sciences. The use of fractal analysis in studies of substance use remains disparate, although notable works have used fractal dimension to map neurological processes altered through substance use (6); explored the relationship between alcohol-related aggression and dopaminergic transmission through fractal analysis (7); and evidenced significant differences in the structural complexity of carotid bodies when deaths were related to opioids (8). However, although fractals have had diverse applications, never has fractal mathematics been applied in qualitative or ethnographic studies of addiction and substance use. Neither have time experience and temporality been seriously theorized in relation to fractals. As this paper demonstrates, however, fractal patterning is evident across many aspects of life for people who use drugs, and particularly in experiences of time shaped by substances.

This paper traces the intimate and intersubjective experiences of people who use heroin and other drugs, many of whom were attempting to negotiate multiple institutions, financial and social precarity, and their own specific reckonings with time and memory. Presenting snapshots of sustained ethnographic fieldwork from two Southeast counties in Scotland, the paper delves into time consciousness in active drug use, illustrated through individual experiences of intoxication. Laura’s fieldwork initially took place over 12 months between 2016 and 2017 in Southeast Scotland, continuing sporadically over several years (5). The first 7 months of research were largely spent in recovery and harm reduction services, including a community abstinence-based recovery group and a third sector needle exchange. During the subsequent 5 months of research, Laura accompanied one scattered group of people who actively used heroin and other drugs in their day to day lives. Primary methods included participant observation, semi-structured interviews, and casual conversation, although the nature of the research necessitated a reflexive, adaptive methodology to account for frequently difficult, unexpected, and ethically complex situations. Findings indicated that time profoundly shaped experiences of addiction and recovery, and that addiction in turn impacted upon senses of time (5).

This paper contends, overall, that a meaningful engagement with fractal theory contains the potential to open up new vocabulary, imagery, and theoretical avenues with which to grasp complex and non-linear time experience. The aims of the paper are, therefore, twofold; to both provide a nuanced ethnographic exploration of substance use time and to propose a reflexive analytical framework for temporal experience through fractals.

## Fractals for an ethnography of time

Mathematician Kenneth Falconer notes the difficulties of giving a precise definition to fractals, arguing that fractals are best understood as exhibiting certain characteristics (10). One common characteristic is the fractal tendency to exhibit recursive, self-similar patterns across multiple scales. That is, fractals often contain nested patterns within patterns, which appear near identical from any perspective. Coral reefs are frequently given as examples of natural fractals, as magnified “parts,” at any scale, broadly resemble the “whole” in appearance and structure. Coral reefs also exemplify exceptional structural complexity that renders their study challenging. Falconer likewise notes that mathematics has often excluded objects deemed too irregular to be examined within the methods and bounds of classical calculus, or described in “traditional geometric language,” but that fractal geometry offers the potential to better grasp and express complexity (9).

Anthropological and mathematical understandings of complexity are admittedly very different, and the term possesses an array of meanings in both disciplines that make analytical use problematic. Time described as “complex” might connote, in a simple sense of the word, the complicated, the intricate, and the obscure, each of which can be used to characterize the temporalities experienced through substance use. Outside of common parlance, however, complexity may also be considered in the mathematical sense, broadly relating to the nature of systems and their relationships with their component parts. As with fractals, however, there is no one definition of complexity in mathematics, the concept instead depending upon the context it is applied in (10). And as also with fractals, anthropology has a rich history of engaging with complexity theory, perhaps most notably within linguistics (11, 12). In contrast with structuralist approaches to language–which broadly hold that “root” oppositional pairs of signifiers give rise to binary metonymic associations, from which spring “trees” of symbolic classificatory systems–post-structuralist models of language consider signifiers as unfixed and operating in a complex network (13). That is, there exists an “indefinite referral of signifier to signified,” in which the meaning of signs is “endlessly deferred” (12, 14). Language and signification are as such composed through the endlessly complex interactions of individual parts, which comprise classificatory systems. Our interest, too, in understanding time as “complex,” lies largely in the intermingling of dynamic, infinitesimal, and ever-shifting components of temporal experience and memory–that is, in the “non-linear relationships between constantly changing entities” (10).

How, then, might time be viewed as complex in a manner accounting for the co-implication of interrelating components? To take the example of substance addiction, it was apparent during Laura’s research that time was often experienced as fractured and continually fracturing, due to specific forms and means of intoxication and overlapping experiences of trauma and loss. Time alternately splintered, slowed, accelerated, and was lost altogether, to produce an overarching sense of time that was composed of shifting and fluid parts, but which could not be reduced to those parts. Senses of fractures and fragmentation in moments of time were often felt to accumulate, interact, and replicate across wider spans of days, months, and years. Complexity arose in the coalescence of conflicting temporalities to forge forceful senses of time as non-linear, recursive, and self-similar across scales: that is, of time as fractal.

## Representing fractal and temporal complexity

**FIGURE 1:**
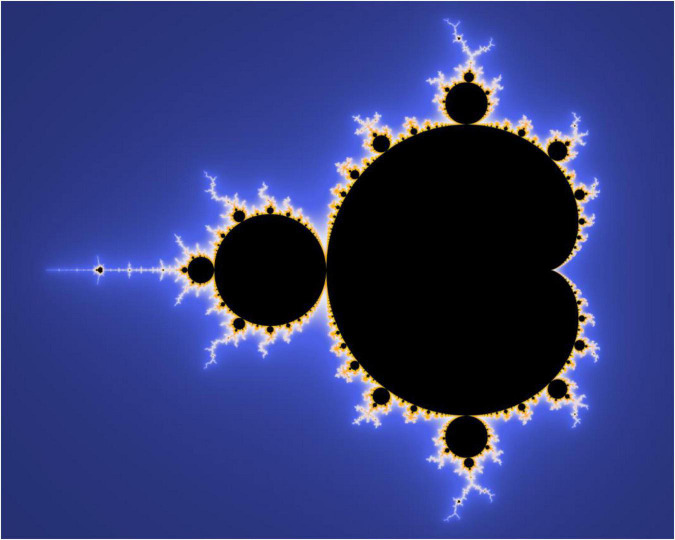
The Mandelbrot set (public domain image).

**FIGURE 2:**
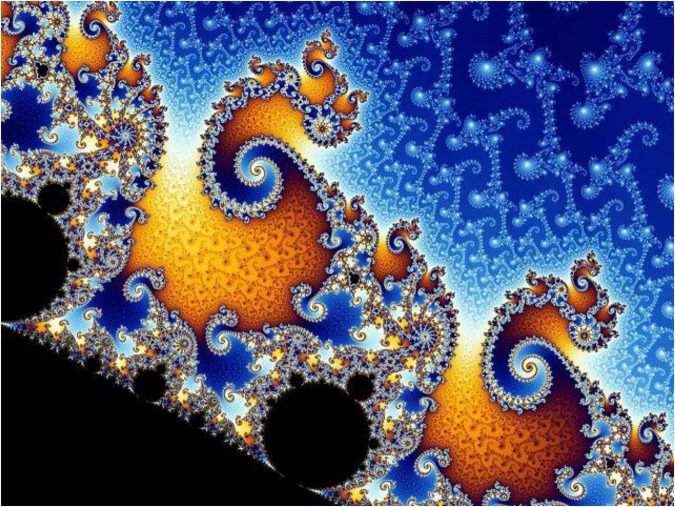
Mandelbrot zoom sequence (Image credit: Wolfgang Beyer at: https://commons.wikimedia.org/w/index.php?search = the + mandelbrot + set&title = Special:MediaSearch&go = Go&type = image).

Most well-known images of fractals, such as the Cantor set, the Julia set, and the Sierpinski triangle, illustrate only coarse approximations of the fractal, which are too complicated to represent visually due to their infinitesimal details. An example is that of the Mandelbrot set (Figures 1, 2), publicized by Benoit Mandelbrot, images of which depict infinitely fine and recursive details at increasing magnifications.

The set somewhat adheres to the fractal principle of self-similarity across scales, as it contains rough copies of itself, and yet each magnification of the set brings new features and “surprises” (15). Mandelbrot himself is noted by James Gleick to have been continuously astonished at the set’s complexity, with Gleick noting that “there were always new kinds of seahorses, new curling hot house species…no part of the set exactly resembles any other part at any magnification” (15). Gleick goes on to describe the set as “a miracle of miniaturization in which every new detail was sure to be a universe of its own, diverse and entire” (15). It was such that prompted Marilyn Strathern to remark that “the whorls and involutions of these self-similar shapes that repeat motifs through any scale of magnification produce the most seductive visuals” (16).

The innate representational challenges of fractal mathematics bear relevance to the difficulties faced in anthropology and other disciplines to reckon with the full complexity and multiplicity of time and temporal consciousness. These challenges are, moreover, keenly felt in describing time experienced through poly-substance intoxication. In striking resemblance to the fractal characteristics outlined above, Laura’s participants emphasized the intricate variety of instances of getting high, which come to take on broad qualities of sameness, or “self-similarity” through time. The highly embodied, individual, and situated nature of polydrug use, however, was ever-difficult to render in words.

### Tracing dust

Among people who actively used substances in the research, there were myriad reasons for getting high, although intoxication was commonly positioned as a means of slowing fast-paced, “chaotic” temporalities, distancing troubling cares and memories, or alleviating feelings of boredom and emptiness. Getting high could also, however, induce desirable senses of emptiness and quietude, in which one was alone with the substance, and foster senses of time as endless and unbounded. People often mixed potent cocktails not limited to opiates, but encompassing alcohol, cocaine, benzodiazepines, and amphetamines, amongst myriad other substances. The effects, temporal and other, were many, often existing on the cusp of purposeful and accidental. Participants found themselves tenuously *pursuing* the non-fatal effects of overdose; that is, uneven loss of consciousness, dissociation, detachment, and a drastic slowing of the central nervous system. The fracturing of time and memory, too, was at times actively sought, with both positive and negative results. A woman named Tamsin, for example, who Laura came to know well over the course of the research, once described the effect on time of being intoxicated on heroin, alcohol, and Valium:

You don’t really feel time passing in the normal way. Have you ever been so drunk, everything’s all mixed up and your memories are shot to shit? It’s like that, sort of. You can’t remember what happened when, and it’s all out of sync, hard to put back in order […] lots of gaps […] It can be nice, to feel like you’re in a dream. Or just forget shit for a bit, ken. But it’s not so nice when people are telling you you did stuff you can’t remember.

When asked if memories that were lost ever returned, Tamsin responded, “Aye. It depends… you vaguely know *something*. It’s like it’s still there, but, like, out of reach.” Some months later, when driving Tamsin to an appointment by car, Laura continued to ask about the impact of multiple substances on experiences of time and the recollection of memories.

It’s carnage, complete carnage, like, I just want to lose myself, forget shit, that’s why I do it. That’s why I take this shit. You don’t notice time at all, I’d say, you’re out of it. It just goes on, and on, and on, and on, ken, one minute it’s ten in the morning, the next minute it’s dark outside and you can’t remember… you maybe do get flashes after, it depends what I’ve been taking, ken, Laura. Heroin won’t…it keeps me straight, that’s not why…but there’s times I want to just get mad with it and I keep going down […] Then, yeah, I’ll take coke and Vallies or I drink, and then I start to lose my, my…ken […] I just shatter myself. It’s exhausting, it’s every day, on and on.

Like others, Tamsin complained that the feeling of being high was difficult, if not impossible, to describe, though her above imaginings of time and memory evoke fractal dissonance, fracture, and incoherence, as with her use of the terms “out of sync” and “shatter.” Time appears to slip away altogether at times, with the potential to be reconstituted in the words and testaments of others. Her initial descriptions evoke a pleasurable, dreamlike passage of time, contingent upon the loss and fragmentation of time-sense and memory, which in turn renders both difficult to trace, interpret, and express. Memories are imbued with an intermittent and almost spectral quality, hovering somewhere between remembering and forgetting, presence and absence. They may be non-sensical and fragmented beyond recall, though without entirely disappearing–instead traces persist almost imperceptibly, like particles of dust in the atmosphere. Tamsin further gestures toward senses of endlessness and paused time in intoxication–time that is at once unending and yet collapsed into the situated experience of getting high. Temporality, in this configuration, is not progressive, but rather has different depths (17). Her descriptions situate the experience within space, indicating a progressive and recursive experience of “going down,” to imply escalating use, and calling up images of fragments and fractures that return in “flashes.”

Much past anthropological work on fractals has concerned the interrelation of fragments and imagined gaps in between, even over that of the fragmentation (16, 18). In her fractal analysis of the Balkans, Sarah Green, following Strathern, posits that relations between entities are formed through the gaps created by “fragments,” as much through the fragments themselves (18). Rather than being empty spaces, gaps paradoxically serve as points of connection. Indeed, Green argues that the constructed fractality of the Balkans at the level of the whole is constituted through the relations between parts and gaps, and their chaotic and recursive “intermingling” (18). In the experiences of people who use drugs, fragmented temporalities at times collide and interweave to produce an overwhelming sense of chaos. The presence and intersection of gaps or missing memories exacerbated such feelings.

In a wider sense, memory and non-memory, fragments, and gaps, interrelated in unusual ways, manifesting in desires for specific states of intoxication. As Tamsin was earlier quoted, people sought to distance certain memories and distressing past experiences through substance use. The desired forgetting of memories was positioned against the happenstance loss of others. In ways not demonstrated through Tamsin’s sentiments, however, memory and experiences could be made proximate by using substances, though rarely in coherent or readily understood forms. Angela Garcia, for instance, envisions heroin use as a means of tending to past experiences of loss and grief in the present (19). Rarely are connections clearly made, however, between heroin and recollections of the past, and only ever partially articulated in “isolated utterances.” Fragments and the gaps between them in the context of heroin addiction therefore shift, conflict, and interact in a complex, dynamic web of memory and meaning. Interpreting these, again, is made problematic by their complexity, irregularity, and ambiguity.

What Garcia also evidences is that the minutiae of the everyday appear disconnected and obscured from broader temporal and affective trajectories (19). Thinking on intoxication, an appearance of fractal self-similarity between smaller scales and wider timeframes is complicated by the impossibility of reassembling the fragments. Veena Das suggests in her discussion of violence that “unlike a sketch that may be executed on a different scale from the final picture one draws, or that may lack all the details of the picture but still contain the imagination of the whole, the *fragment* marks the impossibility of such an imagination” (20). It can be argued, however, that the fragment at least denotes the former existence of a whole. Often as a result of extreme intoxication, time-perception and memory formation cease altogether, and fragments better resemble dust: as Tamsin suggested, somehow perceptible and imperceptible, but not particularly suggestive of a former whole. They cannot be taken separately, but instead blend and mingle together: impossible to return to whatever came before (18).

On appearance, then, memory and time in contexts of intoxication are neither self-similar nor recursive. Switching scales and perspectives, however, it is possible to see that, however, uninterpretable the “dust” and fragments appear, the felt temporal chaos it elicits *is* reflected on wider timescales. Interlocutors continually emphasized the constant fracturing of the everyday, and the spillage of this into longer periods of years–so that fracture, breakage, and distortion came to characterize multiple scales of addictive time. Many of Laura’s interlocutors’ attempts to pursue abstinence and recovery were frustrated by *both* temporal incoherence and the repetitive, cyclical nature of this incoherence through broader time trajectories. Each compounded what Garcia terms a “dynamic of endlessness,” and solidified the perceived inevitability of returning to what had already been lived and would be lived, to quote Nietzsche, “once again and innumerable times again” (19, 21). In turn, the seemingly unending, self-similar nature of addiction was refracted, fractally, in experiences of the high.

## Conclusion

Through explorations of time, temporality, and memory, fractals can provoke new avenues of thought–providing a means with which to better understand, excavate, and portray experiences that sit outside of classical conceptions of linear time, and for which language is often insufficient to grasp. An engagement with fractal theory, as such, not only illuminates ways in which time comes to bear on everyday life across multiple temporal scales, but provides expanded linguistic, epistemic, and hermeneutic frameworks with which to apprehend (mathematically) complex, incoherent, and ineffable experience. Greater attention to time experience in addiction is something that, we propose, should accompany an ethical embracing of uncertainties, gaps, and complexities, and a refusal of over-simplified or generalized portrayals of substance use.

While the focus of this piece was experiences of active drug use and intoxication, the research also highlighted fractal-like patterns in experiences of abstinence, recovery, and relapse. Many participants related time as endlessly repetitive in their attempts to stop using drugs and experiences of relapse, some emphasizing that nesting within each new attempt to recover was the memory of the last unsuccessful attempt, and within it the preceding, and so on. Clinical neuroscience literature attempts to evidence a similar pattern, positing that each attempt at abstinence followed by a return to heroin makes the chances of recovering less and less likely (22). It is our perspective, overall, that fractal theory has rich potential to investigate a range of aspects of addiction; for example, critically interrogating simplistic linear/cyclical framings of recovery, abstinence, and treatment. Fractal analysis could likewise be employed to explore state and institutional treatment of temporal complexity; stigmatizing stereotypes associated with “chaotic” and recursive temporal experience; and the damaging insistence of recovery organizations on service users’ pursuit of a linear timeframe. More broadly, an engagement with fractals underscores the potential of interdisciplinary collaborations, toward a translatory dialectic in ethnographic praxis and participant-led theory.

An orientation to time, overall, aids in tracing the invisible structural, social, and historical processes that operate at the level of everyday experience, as well as the minutiae of such experience. Where time and experience are difficult to trace, express, and make sense of, fractals open up space for analysis without imposing rationality or coherence: one in which time as dust and depth is made possible.

## Data availability statement

The original contributions presented in this study are included in the article/supplementary material, further inquiries can be directed to the corresponding author.

## Ethics statement

The studies involving human participants were reviewed and approved by University Teaching and Research Ethics Committee (UTREC), University of St Andrews. The patients/participants provided their written informed consent to participate in this study. Written informed consent was obtained from the individual(s) for the publication of any potentially identifiable images or data included in this article.

## Author contributions

LR conducted the ethnographic research that informs the manuscript. SD, LR, and HW developed the concept for the manuscript. All authors contributed to the drafting and revision of the work.
